# Medical decision-making in children and adolescents: developmental and neuroscientific aspects

**DOI:** 10.1186/s12887-017-0869-x

**Published:** 2017-05-08

**Authors:** Petronella Grootens-Wiegers, Irma M. Hein, Jos M. van den Broek, Martine C. de Vries

**Affiliations:** 1 Science Communication and Society, Leiden University, Leiden, The Netherlands; 2 Athena Institute for Research on Innovation and Communication in Health and Life Sciences, Faculty of Earth and Life Sciences, VU University Amsterdam, Amsterdam, The Netherlands; 3 Child and Adolescent Psychiatry and de Bascule, Academic Medical Center Amsterdam, Amsterdam, The Netherlands; 4 Department of Medical Ethics and Health Law, Leiden University Medical Center, Leiden, The Netherlands; 5 Department of Pediatrics, Leiden University Medical Center, Leiden, The Netherlands

**Keywords:** Decision-making, Neuroscience, Competence, Children, Adolescents, Brain development, Minors

## Abstract

**Background:**

Various international laws and guidelines stress the importance of respecting the developing autonomy of children and involving minors in decision-making regarding treatment and research participation. However, no universal agreement exists as to at what age minors should be deemed decision-making competent. Minors of the same age may show different levels of maturity. In addition, patients deemed rational conversation-partners as a child can suddenly become noncompliant as an adolescent. Age, context and development all play a role in decision-making competence. In this article we adopt a perspective on competence that specifically focuses on the impact of brain development on the child’s decision-making process.

**Main body:**

We believe that the discussion on decision-making competence of minors can greatly benefit from a multidisciplinary approach. We adopted such an approach in order to contribute to the understanding on how to deal with children in decision-making situations. Evidence emerging from neuroscience research concerning the developing brain structures in minors is combined with insights from various other fields, such as psychology, decision-making science and ethics. Four capacities have been described that are required for (medical) decision-making: (1) communicating a choice; (2) understanding; (3) reasoning; and (4) appreciation. Each capacity is related to a number of specific skills and abilities that need to be sufficiently developed to support the capacity. Based on this approach it can be concluded that at the age of 12 children can have the capacity to be decision-making competent. However, this age coincides with the onset of adolescence. Early development of the brain’s reward system combined with late development of the control system diminishes decision-making competence in adolescents in specific contexts. We conclude that even adolescents possessing capacities required for decision-making, may need support of facilitating environmental factors.

**Conclusion:**

This paper intends to offer insight in neuroscientific mechanisms underlying the medical decision-making capacities in minors and to stimulate practices for optimal involvement of minors. Developing minors become increasingly capable of decision-making, but the neurobiological development in adolescence affects competence in specific contexts. Adequate support should be offered in order to create a context in which minors can make competently make decisions.

**Electronic supplementary material:**

The online version of this article (doi:10.1186/s12887-017-0869-x) contains supplementary material, which is available to authorized users.

## Background

Various international guidelines stress the importance of involving children in decision-making regarding medical treatments and research participation. According to article 12 of the UN Convention on the Rights of the Child, “children shall be provided with the opportunity to be heard in any judicial or administrative proceeding affecting the child directly” [1]. More specific medical guidelines include The Second Directive by the European Parliament and the Council of the European Union, which states “A clinical trial on minors may be undertaken only if [] the minor has received information according to its capacity of understanding” [2]. In addition, many countries have laws specifying at what age children should be involved in decisions about medical treatment or scientific research. In the Netherlands for example, children from the age of 16 may take treatment decisions independently, and children from the age of 12 are allowed to give informed consent for research participation or treatment together with their parents. In the US a minimum age of 7 years old is defined for asking assent (as opposed to legal consent) from children [3]. In the UK, children under the age of 16 cannot be treated without parental consent, unless they prove to be mature according to the Gillick ruling [4].

These laws and guidelines underline the importance of respecting the developing autonomy of children. However, they also show that there is no universal agreement as to at what age it is appropriate for children to be considered competent for decision-making. Empirical evidence demonstrates that children have an emerging competence at a very young age. Weithorn & Campbell found children as young as 9 years old to have the capacity to make informed choices [5]. In addition, some studies conclude that children at age 14 or 15 are as competent as adults [5–7]. A recent study demonstrated that generally children older than 11.2 years may be competent to consent to clinical research [8]. Yet in most countries, children are considered incompetent until the age of 18 or 21, when they officially have reached legal adulthood.

In medical practice it is not clear-cut whether a child of a certain age is sufficiently competent for medical decision-making. Different children of the same age may have a different level of maturity. Young children, who have demonstrated sufficient competence for decision-making in a certain situation, can lack adequate competence in another. Furthermore, children who have shown to be reasonable conversation-partners during their treatment, can (temporarily) be noncompliant in adolescence, as illustrated by the story of Elsa in Table 1. Therefore, in this article we explore a way in which insights in brain development can contribute to insights in decision-making competence of children at various ages.

**Table 1:** The story of Elsa

Elsa is a 16 year old adolescent who was diagnosed with diabetes type I at the age of 4. The first years after the diagnosis, Elsa’s parents did all the diabetes care. They measured blood sugars, and adjusted insulin dose as required during meals, exercise etc. The insulin pump Elsa was wearing had a child safety lock to prevent accidental use by Elsa. Elsa was able to express how she felt about the disease but did not have any influence in the treatment. When Elsa became older, she was very eager to learn about her daily diabetes care. From the age of 7 she was taught how to measure her own blood sugar and what the result meant. From about the age of 8, she could instruct the pump to give the insulin dose needed during meals (as long as her parents had written down in her lunch box how many carbohydrates were in the lunch). At 10, Elsa showed profound insight in how to adjust her insulin pump settings when her blood glucose levels were not optimal. By then, she was so well informed and experienced that she was able to handle her diabetes with her parents only exerting global supervision. When Elsa turned 12 and went to secondary school things changed. She started to exert less self-control. She did not measure glucose levels and did not inject insulin for meals at school. Her school friends were unaware of her diabetes because Elsa did not inform them. Elsa tried to deny her diabetes at school, and often even took off the insulin pump, for example during physical exercise at school. When at the pediatrician’s office, Elsa was always friendly, showing remorse and promising improvement. At age 14 however, she had to be admitted to the Intensive Care Unit because of severe dysregulation of her diabetes and an acute life-threatening situation. At age 16, the same happened after drinking large amounts of alcohol	

### Decision-making competence and capacity

A certain level of competence is required for medical decision-making in order to balance the respect for autonomy with the protection of vulnerable patients [9]. In order to be sufficiently *competent*, one needs to have the *mental capacity* to make decisions, but also should be accountable of the decision in the specific situation. That is, one can in theory have the mental ability to make a reasonable decision, but a certain situation can reduce a person’s competence, e.g. due to stress or peer pressure [10]. *Decision-making capacity* is thus necessary, but not sufficient for being *decision-making competent.*

Decision-making capacity can be defined by four standards: (1) expressing a choice; (2) understanding; (3) reasoning; and (4) appreciation [11–13]. In order to be considered competent to make a decision all four capacity standards should be met [11, 13].

However, decision-making competence is not an on-or-off phenomenon [14], but is relative to the specific decision in the specific situation [14, 15]. Furthermore, certain diseases, medical as well as mental, can affect competence, either temporarily (e.g. when a patient loses consciousness) or in a chronical manner (as is the case in progressing Alzheimer’s disease [16]).

Miller has proposed a model on children’s capacity in which initial predisposing factors are identified, followed by four groups of factors that influence decision-making competence, namely child, parent, clinician, and situational factors [10]. Predisposing factors include the discussed cognitive development, as well as experience. Factors related to the child are personality [17], and emotional state of the child that can affect capacity and serve as a spotlight or motivator for information and preferences [6, 17, 18]. In addition, disease severity can affect understanding, as well as retention of information and reasons to consent [19]. Parent and clinicians can influence the child’s competence with their attitude towards the child and the attention and support provided in the decision-making process [6, 17, 20]. Finally, situational factors as the type and complexity of the decision, the setting and time constraints play a role [10].

In Miller’s model, (cognitive) development is thus an important predisposing factor for decision-making competence in children. As children grow older, their capacities to comprehend information and therefore competence to make a decision increase. Therefore, insight in the development of various abilities related to medical decision-making may contribute to understanding at what age children could be considered decision-making competent.

### Aim

We believe that the discussion about decision-making competence of minors can greatly benefit from a multidisciplinary approach, as the issue has many aspects. We reviewed the evidence emerging from neuroscience research concerning the impact of developing brain structures on children’s decision-making capacities and competence. We subsequently combined insights from neuroscience with various other fields: psychology, decision-making science, ethics and medical practice. It is not our aim to quantify specifically at what age exactly children should be considered decision-making competent, but rather to contribute to insights on how to deal with children in medical decision-making, and to add to the general discussion on children and decision-making.

In this paper, we will discuss the aforementioned four standards of medical decision-making capacity as defined by Appelbaum and Grisso [13]. We will discuss the development of the various skills and abilities that are required for each standard according to Appelbaum and Grisso [13], as well as describe the brain areas that are involved in these skills. Relating brain areas, development and decision-making abilities can contribute to an understanding of child behavior and competence. However, we will only be able to provide a simplified insight in the neuroscience background, as each ability requires the contribution of numerous brain areas and structures and we aim to keep this discussion readable for clinicians without a background in neuroscience. For a more elaborate overview of brain structures involved in decision-making, we want to point the reader to the paper of Rosenbloom et al. [21].

In addition, we will discuss what happens in the brain during adolescence and how this influences decision-making. Adolescents often seem to have a reduced ability to make reasonable decisions [22, 23], and this phenomenon can be related to the developmental events happening in the brain during this period. The paragraph on adolescents will enlighten why many adolescent patients will consent to treatment in the clinic but do not do as asked when they return to normal day-to-day life, such as in the story of Elsa (Table 1).

### Development of abilities and brain areas related to the four capacity standards

The four standards of medical decision-making capacity will be discussed in association with neurological skills. In this section, the main course of development is discussed, a more detailed discussion of the neurological skills and related brain areas is provided in the Appendix (Additional file 1).

#### Expressing a choice

The first and least rigorous standard for decision-making capacity is the ability to express a choice. This standard implies that someone can communicate a preference of treatment or research participation, which is legally restricted to spoken or written language. The required neurological skill for this standard is being able to **communicate**, either in spoken language or nonverbally [13, 24]. Nonverbal communication can be used as an indication of dissent or of implicit consent, but not as a legal form of consent. Therefore this capacity is mainly related to verbal language development, which initiates in early childhood. From the age of 5, children have reasonable understanding of language, with refinement thereof continuing to the age of 9 and further throughout adolescence [25].

#### Understanding

The second standard requires the ability to understand the information provided about the proposed medical treatment or research and comprehending the fact that a choice needs to be made. Understanding requires a combination of neurological skills [13, 24]: One first needs to have sufficient **intelligence** and **language** proficiency to process the information. Further, one needs to be able to **orient** and direct **attention** towards the information. In addition, understanding requires **memory** and **recall** skills, in order to process and integrate information beyond the short-term moment. The foundation for these skills is laid down in the first year of life. Maturity in orienting and attention develops around the ages of 7–10 [26–28]. During childhood the ability to remember information and the amount that can be remembered develops. Memory specifically increases between the ages of 6 and 12, and then goes on to slightly increase during adolescence [29, 30]. Children at the age of 10–12 appear to have recall abilities compared to adults [31–33].

#### Reasoning

The third standard is that, next to understanding the factual information, someone should be able to reason about risks, benefits and possible consequences of the treatment or research options presented [11, 13, 24]. This standard is a step further from factual understanding and requires the ability for logical **reasoning** and **weighing risks and benefits**. Children at the age of 6 to 8 already demonstrate the ability for logic reasoning [34, 35]. Between the ages of 8 and 11, children’s reasoning skills improve significantly, mainly due to improved use and access to their own knowledge [36]. Complex reasoning about alternative causal relations needs more time to develop, in adolescence is has become more accurate, but even adults often make mistakes [34]. Risk identification develops strongly between the ages of 6 and 10 [37]. Adults are better in identifying risks than children and adolescents but not in identification of benefits [38]. In addition, as will be discussed later in this paper, even though risk identification is mature in late adolescence, the way people of this age will deal with risks differs from that of adults.

#### Appreciation

The strictest standard of decision-making capacity is appreciation. The appreciation of the nature of a situation implies that someone will not only understand the various options, but also the relevance of these options for the personal situation. In order to appreciate the situation and personal relevance of the decision at hand, one needs to have the ability of **abstract thinking**, which includes being aware that others have a mind of their own, which is called **theory of mind** [12, 13]. Abstract thinking, about things that are intangible, is necessary to understand the consequences of a decision. There are many different skills and brain areas involved in this skill. Between the age of 3 and 4, children already start to recognize their own beliefs and desires, which contribute to the development of personal norms and values, and start to understand how these influence their actions [25, 39, 40]. Improvement of the efficiency of working memory with age further increases the ability to think about abstract and hypothetical things, situations and norms and values [34, 41].

### Model

**Fig. 1:**
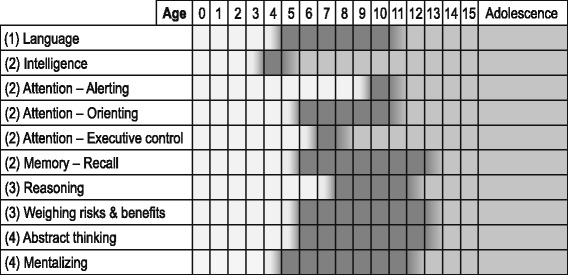
Development of decision-making capacity. In this figure the critical developmental period (*darkest*) for each of the discussed abilities is reflected. Each box indicates 1 year of life: e.g. the box under 0 indicates the period between birth and reaching the age of 1. The development of each ability starts at an early age, and continues to mature to a small or larger extent into adolescence or even beyond

Below the discussed abilities and their developmental trajectories are visualized in a model (see Fig. 1). This overview shows that the necessary abilities and relating brain areas do not develop synchronically; some aspects of capacity are mature much earlier than others. This illustrates that decision-making competence is not an on-or-off concept, but rather a growing skill with age.

### Translation of the model into clinical practice

The four capacity standards can be measured in clinical practice with the use of the MacArthur Competence Assessment Tool (MacCAT) [13], which is validated and used among adults. Results of a recent study on children’s competence to consent to clinical research showed that the MacCAT could also be validly and reliably used in children. The MacCAT-CR was studied in a population of pediatric patients between 6 and 18 years of age [8]. The study demonstrated that age limits for children to be deemed competent to decide on research participation could be estimated as follows: children of 11.2 years and above generally appeared to be competent, whereas children of 9.6 years and younger were generally not competent. A change-over occurred between 9.6 and 11.2 years, and the cross-over point was estimated at 10.4 years [8]. In the same study, the four domains representing competence in most jurisdictions (understanding, appreciation, reasoning, and expressing a choice) appeared to constitute a single trait in children. These results correspond well with the model in Fig. 1. Below the age of 10, too many abilities are still in their (early) development and overall competence cannot be expected. However, as discussed, the cut-off age of 11.2 does not automatically imply competence for any decision in any situation. Rather, this age serves as an indication at what age competence might be expected given favorable environmental factors. In addition, an important influence on competence is the rise of adolescence, which is accompanied with very specific events in brain development.

### Adolescence and decision-making competence

The demonstrated model might suggest a linear pattern in development and a corresponding linear increase in decision-making competence with age. However, due to differences in cross-talk between the various brain structures over the course of brain development, competence might fluctuate. A period in which this is especially pronounced is adolescence. In this period, great changes and developmental leaps take place in the brain, which can have a profound effect on decision-making competence.

Adolescence is a period associated with a number of health issues and increased mortality [42, 43]. Adolescents often have increased appetite and therefore a change in diet; in addition adolescence is typically the time where tobacco addiction initiates and a time of emerging alcohol and substance (ab)use [42, 44]. Further, for chronically ill children, this is a time where the disease management approach can change, sometimes creating risky of even life-threatening situations, as illustrated in Table 1. The increased mortality seen in adolescence is mostly associated with risky behavior, sensation-seeking and peer influences affecting decision-making [43].

Adolescence starts around the age of 12 and the neurologic developments initiated can continue into early adulthood [7, 45]. The brain in adolescence differs significantly from the brain in childhood and adulthood [45–48]. To gain more insight in the effect of adolescence on decision-making, it is important to have an understanding of this period. The most significant changes in the brain are associated with processing rewards and risks, self-regulation, and the effect of peers on decision-making. These neurologic changes affect decision-making in general and, depending upon context, can affect medical decision-making to a certain extent as well.

### Risk, sensation-seeking and self-regulation

Adolescents are prone towards increased risk-taking and this is associated with the development of a number of brain-structures. Two brain systems are especially important: the prefrontal cortex (PFC), which is the control system; and the ventral striatum, the reward system. The control system is involved in impulse control, the ability to stop a certain urge or action, and thus involved in self-regulation. The ability for self-regulation develops strongly from the age of 12 until the age of 18 [45], but continues to improve into early adulthood [7]. In addition, the prefrontal cortex also performs better at other functions that require control, such as planning ahead, weighing risks and benefits and in processing complicated decisions. The cross-talk between the control system and the reward system and associated emotional regulation is not fully developed before early adulthood [7]. This means that even though an adolescent can have intellectual maturity, this does not automatically imply the presence of emotional and social maturity [7, 47].

The reward system involves a structure that creates dopamine in response to rewards. Dopamine gives a feeling of pleasure, which can lead to learning and the urge to repeat the experience. During adolescence, the reward system becomes hyperresponsive, the dopamine response to a reward is much higher [49]. This is associated with increased reward-seeking and sensation-seeking [48–50]. The increased responsiveness of the reward system even applies to small rewards, making the positive effect of a small ‘success’ of a decision more pronounced for adolescents than for children or adults [49]. Thus in a dilemma in which there is a small chance of a reward, this reward can be attributed such a high value that the situation is no longer perceived as a dilemma by the adolescent and there is only one path to choose [22].

The development of the control and the reward systems do not follow a linear pattern, The last brain areas to mature are those involved in executive function and attention, located in the PFC [51]. Based on structural brain development research, there appears to be a ‘mismatch’ between the development of various regions, specifically the amygdala and the PFC. The amygdala, responsible for emotion processing and input in the reward system, starts to mature in late childhood and stabilizes at mid- to late adolescence [52]. However, the PFC starts to mature in early adolescence and it is not until young adulthood that this area is mature. In addition, the nucleus accumbens in the ventral striatum, appears to develop early in some and later in others, which might explain a ‘mismatch’ in some adolescents [52].

Thus, the control system (PFC) develops slowly, even into early adulthood whereas the reward system (amygdala and possibly nucleus accumbens) already changes in early adolescence [7]. This nonlinear development accounts for the risky decisions often observed in adolescents, such as binge drinking or drunk driving [22]. This is not to say that adolescents are incapable of estimating risks or making responsible decisions. Evidence from laboratory experiments demonstrates that adolescents have a decision-making capacity similar to adults [7, 44, 47, 53]. Adolescents thus have better insight in decision-making than children do, consistent with our proposed model. Yet do they end up in precarious and risky situations and their behavior is often not consistent with their capacities.

This inconsistency can be explained with the distinction between ‘hot’ and ‘cold’ contexts. An emotional context is called a ‘hot’ situation, whereas in ‘cold’ situations, decisions are not or only minimally emotionally loaded [22]. When emotions play a role in a situation, this can significantly influence the decision-making process and outcome [44, 54]. Whether a situation is hot or cold is not predefined: it can vary per individual to what extent a context is perceived as emotionally loaded [48]. Research has shown that during adolescence, risk-taking in decisions in cold situations is similar to that of children and adults [48]. However, when in a hot situation, risk-taking is increased, affecting decision-making severely [7, 44, 48]. This explains the often risky decisions that adolescents make, seemingly only thinking about short-term rewards, even though afterwards they can reasonably assess their leap in judgment.

One particular type of emotionally loaded situation is the presence of peers. As adolescence is essentially a process to develop the capacity to navigate the social landscape, social cues become increasingly important [53]. During adolescence, the acceptation by peers becomes an important purpose in everyday life and guides decision-making [55]. Correspondingly, the ability to understand the perspective of another person and predict that person’s behavior increases [48]. As discussed, this ability for mentalizing develops until late adolescence and it modulates decision-making. In addition, self-awareness increases during adolescence [55].

Accordingly, decision-making in the presence of peers is substantially different from individual decisions [56]. When with peers, the brain sensitizes even more towards rewards and possible rewarding outcomes are higher valued. The adolescent can show an adequate understanding of the situation and its risks involved, but the developing control system can become overruled by the emotional cues in this ‘hot’ context [47]. As a result of the hot context adolescents are more prone towards making risky decisions, even when only a small reward can be expected [43]. This also explains why adolescents’ risk-prone tendencies are mostly observed in group situations, especially when there is a certain form of excitement present (‘hot’) [22].

### Strengths and vulnerabilities of adolescents in medical care

The developing brain in adolescence thus leads to lower cognitive control and leaves adolescents more prone towards risk-taking, especially when together with peers. These characteristics can affect decision-making competence during adolescence. The competence of adolescents to make a decision can vary per situation. Some medical decisions can be considered ‘cold’, with minimal influence of social or emotional factors [7], providing a good context for a competent decision. Treatment and research decisions are generally not impulsive decisions, and a certain amount of time for consideration is provided. This will reduce impulsive and unreasoned decisions in adolescents [47]. However, this does not mean that an adolescent will necessarily live up to the decision in the long run, as context might change. For example, a diabetes patient can be very aware of the benefits of a regular and structured diet and discuss this wisely in a hospital setting. However, living up to the treatment pattern can be much harder when the same person is with a group of friends who decide to skip class and go for a snack. Now the context of the decision turned into a hot, peer-influenced and exciting situation, which affects the decision-making rationale and possibly the outcome, as also illustrated in the example in Table 1. Some adolescents are more susceptible to such an effect than others, and thus the outcome of the dilemma is not necessarily the same for each young patient, making practice very unpredictable.

Especially in treatment situations, adolescents can demonstrate this type of seemingly decreased competence for responsible decisions [42]. Short-term rewards become more important than long-term rewards, even when choosing for an immediate reward can mean a loss on the long-term [48, 53]. This can make it complicated to stick to a healthy lifestyle or treatment pattern, which usually does not deliver immediate rewards, but is meant to increase long-term health. Another factor playing a role might be the expectation of the long-term reward. It appears that adolescents over-estimate their risk of dying soon [57]. This over-estimation of a chance on a short life automatically diminishes the value of any long-term rewards, as the chance of living long enough to receive the reward is considered relatively low.

Although these characteristics render adolescents more vulnerable towards risky situations and their consequences, they also are an important aspect of developing into an adult. During adolescence, the brain shows a high amount of plasticity, resulting in vulnerabilities, but also in opportunities [43]. The sensitivity to rewards together with increased value of social cues creates a perfect situation for learning new skills that are important to function in a social context [53, 55]. Adolescents can learn very quickly and can sometimes even outperform adults when it comes to problem-solving and creativity [53]. In addition, adolescence is a time in which health behavior can be stimulated to consolidate, or when behavior can easily be altered, if the adolescent is motivated to do so [42]. Therefore, adolescence offers an opportunity to target health behavior and disease management and teach the brain new behavior [42, 58].

## Discussion

In this paper we have addressed the complexity of assessing competence in minors and analyzed the neurological development of decision-making capacities based on the four standards from Appelbaum et al.; expressing a choice, understanding, reasoning, and appreciation [13]. The development of the brain demonstrates a nonlinear pattern and therefore decision-making competence does not increase in a linear fashion with age. Based on our model, it might be expected that children around the age of 12 may already have the competence to make medical decisions. However, this age coincides with the onset of adolescence, which is associated with altered decision-making patterns. Adolescents are prone towards increased risk-taking, especially in emotional situations and when with peers. This affects their decision-making competence, mostly in ‘hot’ or emotional situations, such as compliance decisions in everyday life at school, but less so in ‘cold’ situations such as deciding upon treatment in the hospital. As a result, decision-making competence in adolescence can vary greatly between moments and contexts, as was illustrated by the story of Elsa (Table 1). It is thus complicated to pinpoint a certain age at which a child should be considered fully competent to make medical-decisions based on brain development. Even more so since brain development can vary between individuals and gender.

In addition, in this paper we mainly discuss the neurological background of decision-making competence, with the aim to contribute to insights about the age at which the brain is mature enough to be capable of making a decision. However, mature neurological capacity does not automatically mean that a child is competent for any medical decision. There are many factors that influence decision-making competence, either temporarily or chronically, as illustrated by the model of Miller on children’s capacity, describing the predisposing factor of cognitive development, and in addition groups of factors revolving around the child itself, its parents, the clinican, and situational factors. It thus appears impossible to define a cut-off point at what age all children should be presumed competent to make medical decisions based on neuroscience. Nevertheless, based on empirical research, indications for a just age limit for alleged competence to consent in children were estimated. In the clinical research context, children of 11.2 years and above were generally competent. In the treatment context initial indications point into the direction of comparable age limits for alleged competence, around the age of 12, but more research is needed to confirm these findings.

The confirmed potential for competence, combined with the influence of other factors affecting competence, led to the recommendation of a double consent procedure (child and parent) for minors from the age of 12 until 18. Taking into account that parents are generally provided with the legal authority to raise their children, they are assigned with rights and responsibilities. A double consent procedure could achieve an equable consideration between the legal position of the child and that of the parents. A double consent procedure will do justice to both developmental aspects of children and the specific characteristics of the parent–child dyad. The parental role offers extra protection by creating the context for the child’s competent decision-making and by facilitating the child’s long term autonomy. In general, the perspective and attitudes of the adults (both parents and clinician) towards the child may be an important predisposing factor in order to stimulate the highest competence in the child [59]. How adults in the current social climate view minors, can affect whether they live up to their potential. Often children are considered merely on their way to adulthood, but not yet there, This might imply that they are ‘less’ than an adult and are incapable of understanding or forming opinions, let alone making decisions. When children are viewed this way, they will not be informed adequately and will not be supported optimally to take a role in decision-making that does justice to their potential. In order for children to be optimally comptetent, it is important for the involved adults to be aware that children have their own characteristics and perspectives, that are as valuable as (but not necessarily similar to) those of adults, and that they are informed and supported accordingly. This issue will be addressed in more depth in an upcoming paper by the authors (manuscript in preparation).

In accordance with the development of decision-making capacity, and out of respect for children’s autonomy, children should be increasingly informed and involved in the decision-making process [5, 60, 61]. Attention should be paid to providing the child with adequate information, as decision-making competence is *‘only as good as the provided information*’ [14, 20]. This means that the information supplied needs to be adapted to the child’s level of communication and understanding, for example by providing separate sheets for the child and offering oral explanations [60, 62]. As long as there is no adequate information, it is certain that children cannot meaningfully be involved in the decision-making process [9, 59, 63–65].

## Conclusion

Currently, medical laws and regulations reflect the belief that child development influences children’s decision-making processes to the extent that age limits are presented at which children are deemed incompetent or competent. In problematic cases, child psychiatrists and –psychologists are consulted to assess the decision-making capacities of a child, the clinical operationalization of the legal concept of competence. In this article we adopt a perspective on such competence assessment that specifically focuses on the impact of brain development on the child’s decision-making process. Taking this perspective opens up the opportunity to implement results from an emerging field in neurobiological research on how developing brain structures may affect a child’s decision-making capacities. The insights provided in this paper are intended to aid insight in the practice of dealing with minors in medical situations, and to stimulate further discussion about decision-making capacity and competence in children.

In neuroscience, changes in brain structures have been detected that are related to changes in decision-making capacities. The authors are aware that this is a rapidly developing field, that is currently just starting to gain knowledge about the specific development of these abilities in the brain, with many questions left to be answered [18]. As neuroscience is a relatively new and developing science, this paper only provides initial insight in the issue, but evolving neuroscience will lead to further insights.

## Summary

Various international laws and guidelines underline the importance of respecting the developing autonomy of children. However, they also show there is no universal agreement as to at what age children are considered competent for decision-making. In this article we adopt a perspective on competence that specifically focuses on the impact of brain development on the child’s decision-making abilities. Neuroscience research is related to the 4 capacities required for medical decision-making, which are communicating a choice, understanding, reasoning, and appreciation. Based on this approach it can be concluded that at the age of 12 children may have the capacity to be decision-making competent, given favorable environmental factors. However, this age coincides with the onset of adolescence. Early development of the brain’s reward system combined with late development of the control system diminishes decision-making competence in adolescents in specific contexts. We conclude that even adolescents possessing capacities required for decision-making, may need support of facilitating environmental factors.

## Additional file

Additional file 1: Appendix [66–88]. (PDF 692 kb)

## Abbreviations

ACC: Anterior Cingulate Cortex
DLPFC: Dorsal Lateral Prefrontal Cortex
LC: Locus Coeruleus
OFC: Orbitofrontal Cortex
PFC: Prefrontal Cortex
