# Supplementary material for: Medical decision-making in children and adolescents: developmental and neuroscientific aspects
Source: BMC Pediatr. 2017 May 8;17:120. doi: 10.1186/s12887-017-0869-x (PMC5422908; doi:10.1186/s12887-017-0869-x)
Supplement: Additional file 1: — Appendix [66–88]. (PDF 692 kb) [file 12887_2017_869_MOESM1_ESM.pdf]

Appendix

Development of abilities and brain areas related to the four capacity standards.

The four standards of medical decision-making capacity will be discussed in association with neurological skills and brain areas. In order to aid the reader, an overview of the location and function of the discussed brain areas can be found in figure 2.

[Figure 2]

1. **Expressing a choice.**

The first and least rigorous standard for decision-making capacity is the ability to express a choice. This standard implies that someone can communicate a preference of treatment or research participation, which is legally restricted to spoken or written language. The required neurological skill for this standard is being able to **communicate**, either in spoken language or nonverbally [13, 24]. Nonverbal communication can be used as an indication of dissent or of implicit consent, but not as a legal form of consent. Therefore this discussion will focus on verbal language development.

Spoken language develops in close relation to gestures, and becomes more dominant on its own in early childhood [26]. At age 4, children are already capable of producing a substantial degree of language proficiency and an extensive vocabulary [26, 66].

By the age of 6, the language areas of Broca and Wernicke are already active [66, 67], but the connections between the different regions still need to mature. Children use different pathways within these brain areas than adults, as they need complementary structures to support the language processing in the still immature pathways [68]. During language development, the various areas are alternating between increased and decreased involvement [66]. At age 5, children can produce quite complex sentences and have a reasonable

understanding of language [25]. Between the ages of 5 and 9, grammar, vocabulary and pronunciation improve significantly, and this refinement continues throughout adolescence .

2. Understanding.

The second standard requires the ability to understand the information provided about the proposed medical treatment or research and comprehending the fact that a choice needs to be made. Understanding requires a combination of neurological skills [13, 24]: One first needs to have sufficient **intelligence** and **language** proficiency to process the information. Further, one needs to be able to **orient** and direct **attention** towards the information. In addition, understanding requires **memory** and **recall** skills, in order to process and integrate information beyond the short-term moment.

Intelligence, although not an uncommon concept, is hard to define, as there are many different psychological perspectives [66]. For the purpose of assessing decision-making capacity it suffices to state that the child needs to have a certain baseline of thinking skills, processing capacity, and creativity; a low intelligence might hinder understanding and further processing of information. Intelligence consists of various skills and therefore numerous brain areas, as well as connections between those areas play a role in maturing intelligence. Various IQ tests are available to measure someone's intelligence from the age of about 2.5 years old [25]. IQ scores become relatively stable at the age of 4, but the IQ of an individual may still increase or decrease over time as a result of education and environment [25]. IQ scores could be used in the determination whether someone is sufficiently intelligent to make a decision, when this is uncertain.

Attention is necessary to identify the information that should be processed and the context of this information [18]. There are three skills involved in attention: alerting, orienting, and executive control [27, 69, 70]. Although they work together, these three skills

develop at different speed independent of each other and are separate systems in the brain [27, 71].

Alerting is simply the ability to maintain an alert and conscious state and direct attention to incoming stimuli [27, 28, 70]. The brain areas associated to alerting are mostly located in the right hemisphere, more specifically in the right frontal cortex and the right lateral parietal cortex [72]. The neuromodulator norepinephrine secreted in the locus coeruleus influences a number of areas in the frontal cortex and parietal areas, stimulating processes required to alert [70]. The ability to alert develops relatively late in life: it emerges at the age of 12 months, but continues to develop until the age of 10 and beyond [27, 72].

Orienting means to guide attention towards a certain stimulus, such as looking in the direction of a person entering the room [69]. There are two main brain systems involved in orienting: a dorsal system for visual-spatial input, and a ventral system for bottom-up reorienting to shift orientation [70]. The ability to orient is fully developed at late childhood, with the evidence for maturity varying from the ages of 6 to 10 [27, 28].

Executive control is necessary to focus and to prevent distraction by other thoughts or irrelevant information, such as a song playing in the background. The Anterior Cingulate Cortex (ACC) plays an important role in top-down control in the brain, together with the Medial Frontal Cortex [69]. It is thought that there are two separate executive networks; the first one is the cingulo-opercular system which focuses on maintaining a stable task performance. The second network, the frontoparietal system initiates and adjusts control, therefore influencing task initiation and task switching [73]. Both systems consist of a number of different brain areas contributing to executive control. This ability for executive control is already present at a young age; even children of 3 years old can inhibit irrelevant information and focus on a specific stimulus [71]. For relatively simple situations, the ability to control attention conflicts rapidly develops and becomes stable after age 7, while the skill to control

complex situations continues to develop until the ages of 10 to 15 [26-28]. In addition, early education and training can influence executive attention skills as can environment during development [72, 74].

Memory and recall are essential for understanding information, as they are involved in the processing of that information. Information can be retained for a short moment in the short-term memory [26], which is useful for instant recall and to process the information before it is stored more permanently. When information is actively processed, it will proceed to be stored either for an intermediate-term or for the long-term. Intermediate-term storage is for temporarily relevant information (e.g. street names of the neighborhood where you live, or information on a treatment procedure). Long-term storage is (semi)permanently, for information such as date of birth. In order to access the stored information, it can be **recalled** and used in the working memory [26, 66]. An important brain structure in creating new memories is the hippocampus, which is involved in encoding and consolidation of information. In addition, the amygdala plays a role in memory for emotional information. The hippocampus and related brain systems are already in place before birth, but rapidly develop during the first year of life and continue to improve during childhood [26].

During childhood the ability to remember information and the amount that can be remembered develops. The way in which children use their memory and in which they recall information changes during this development, leading to more accurate and more efficient storage [29, 31]. In addition, as more experiences are stored, memory can be used better to interpret new information [18]. Children are able to **recall** information from memory at an early age, but with time the ability increases and children can provide more information and details about a certain event [75].

Memory specifically increases between the ages of 6 and 12, and then goes on to slightly increase during adolescence [29, 30]. Short-term memory develops in a linear fashion

with age and becomes stable after the age of 11 [30, 76]). Long-term memory develops very rapidly between the ages of 6 and 8, then from the age of 9 it develops more slowly, in order to reach a stable point at the age of 12 [30, 76]). The two types of memory thus develop in a different pattern, but reach maturity at the same age. In addition, studies on retrieval tasks demonstrate that at the age of 10-12, children appear to have recall ability comparable to adults [31-33].

3. Reasoning.

The third standard is that, next to understanding the factual information, someone should be able to reason about risks, benefits and possible consequences of the treatment or research options presented [11, 13, 24]. This standard is a step further from factual understanding and requires the ability for logical **reasoning** and **weighing risks and benefits**.

Reasoning is the process by which information that we possess is used to create new insights about information that we do not have. Three systems in the prefrontal cortex that are connected to each other play a role in reasoning: the orbitofrontal cortex (OFC), the ACC and the dorsal lateral prefrontal cortex (DLPFC) [21]. The OFC plays a role in reasoning for a decision related to rewards, punishments and emotions. The ACC is involved in reasoning for complex decisions, such as when there are conflicting options. The DLPFC can integrate multiple sources of information, which is important for more complicated reasoning, using working memory and emotion processing.

There are two main types of reasoning: deductive, or logical reasoning, and inductive, or probabilistic reasoning [66]. Deductive reasoning is based on logical premises, for example: if it rains, the grass becomes wet > it rains > so the grass is wet. Inductive reasoning is based on the likelihood that something will happen, for example: I have seen many swans but never a black one, so black swans probably do not exist. These two types of reasoning are based on different processes in the brain, with different underlying networks [77]. Inductive reasoning

is performed by brain areas involved in recall and evaluation of knowledge on the world, necessary to assess outcome probability. Deductive reasoning, relies on a logic-specific neural network that holds rules for deduction.

There is evidence that people use different reasoning approaches for the same task, depending on working memory strength. When someone has a strong working memory, a more deductive approach will be used, whereas in case of weaker working memory efficiency, a person will rely more on probabilistic information [34, 78]. In addition, the preference for either logic or intuitive reasoning shifts with age [79]. Young children prefer logic reasoning and may even outperform adults in deductive tasks. From adolescence on, intuition starts to play a bigger role in reasoning, sometimes leading to logical fallacies, but in general improving most reasoning outcomes [79].

Children at the age of 6 to 8 already demonstrate the ability for logic reasoning [34, 35]. Around the age of 8 or 9 children will understand the differences between deductive and inductive reasoning and guesses [80-82]. Then, between the ages of 8 and 11, children's reasoning skills improve significantly, mainly due to improved use and access to their own knowledge [36]. In addition, children's insights in good and bad reasoning and thinking quality improve between the ages of 6 and 10 [83]. Complex reasoning about alternative causal relations needs more time to develop, in adolescence it has become more accurate, but even adults often make mistakes [34].

Weighing risks and benefits of various decisional options requires identification of risks and benefits, and the ability to attribute value to them. The ACC plays a role in evaluating risks, uncertainty, and likelihood of success of a plan [84]. The ACC works together with the OCF , which is involved in evaluating the reward and related emotions of certain outcomes and is related to other brain areas involved in emotional processing [21].

In a study with children between 6 and 10, it was found that older children were able to identify more risks for a hypothetical situation than younger children [37]. However, there was no difference between younger and older children with respect to the overall risk rating of the situations [37]. Adults are better in identifying risks than children and adolescents but not in identification of benefits [38]. So even though with increasing age the identification of risks in a situation improves, this does not necessarily mean that the situation will be weighed differently. Risk perception can be influenced by emotional factors, such as feeling vulnerable or feeling safe, and cues from others in the situation [85]. In addition, as will be discussed later in this paper, even though risk identification is mature in late adolescence, the way people of this age will deal with risks differs from that of adults.

4. Appreciation.

The strictest standard of decision-making capacity is appreciation. The appreciation of the nature of a situation implies that someone will not only understand the various options, but also the relevance of these options for the personal situation. In order to appreciate the situation and personal relevance of the decision at hand, one needs to have the ability of **abstract thinking**, which includes being aware that others have a mind of their own, which is called **theory of mind** [12, 13].

Abstract thinking, about things that are intangible, is necessary to understand the consequences of a decision. There are many different skills and brain areas involved in this skill. Thinking about situations that are not present in the current reality requires creating a mental representation [34]. Subsequent manipulation of this mental representation can be used to think about the consequences of various options, e.g. compare burden and benefits of one treatment with another. Between the ages of 3 and 4, the ability to manipulate a mental model increases significantly [34]. Improvement of the efficiency of working memory with age, necessary to hold the mental model, further increases the ability of abstract thinking [34, 41].

In addition, experience and knowledge play a role in hypothetical representations. The ability to create mental models is also closely related with understanding the perspective of others, as conceptualized in 'theory of mind'.

Theory of mind is the ability to understand the perspective or mental states of others and own-mental states [26, 66]. The terms **theory of mind** and **mentalizing** are often used interchangeably, while strictly spoken the first indicates a more factual comprehension and the latter an affective understanding. Mentalizing about others means an understanding that someone has another mind and understanding how someone will feel in a hypothetical situation, or someone else's intentions or knowledge. Mentalizing about oneself is understanding oneself, being aware of own thoughts, and cognitive processes. This skill requires a number of brain structures, such as memory, emotion, emotion regulation, empathy, language and executive functions [21, 86, 87]. Mentalizing requires the ability to 'decouple' thought from reality: one needs to be able to think about a situation, rather than to respond to current sensory input from that situation itself. This decoupling involves the medial prefrontal cortex, which can respond differently between physical responses to a situation and mental representations of that situation [39]. In addition, the OFC is involved in mentalizing, as it plays a role in emotional processing and affects the ability for empathy [21]. Therefore, the development of mentalizing is a process that takes a long time as all involved brain regions need to mature and not do so in a synchronically fashion [87]. Already at elementary school age, children are developing a theory of mind [88].

Between 3 and 4 years old children start to recognize their own beliefs and desires, which contribute to the development of personal norms and values, and start to understand how these influence their own actions and how the beliefs and desires of others motivate the other person's actions [25, 39, 40]. Mentalizing capacity continues to develop at least until the age of 11 and refines until the age of 16 [40, 86].

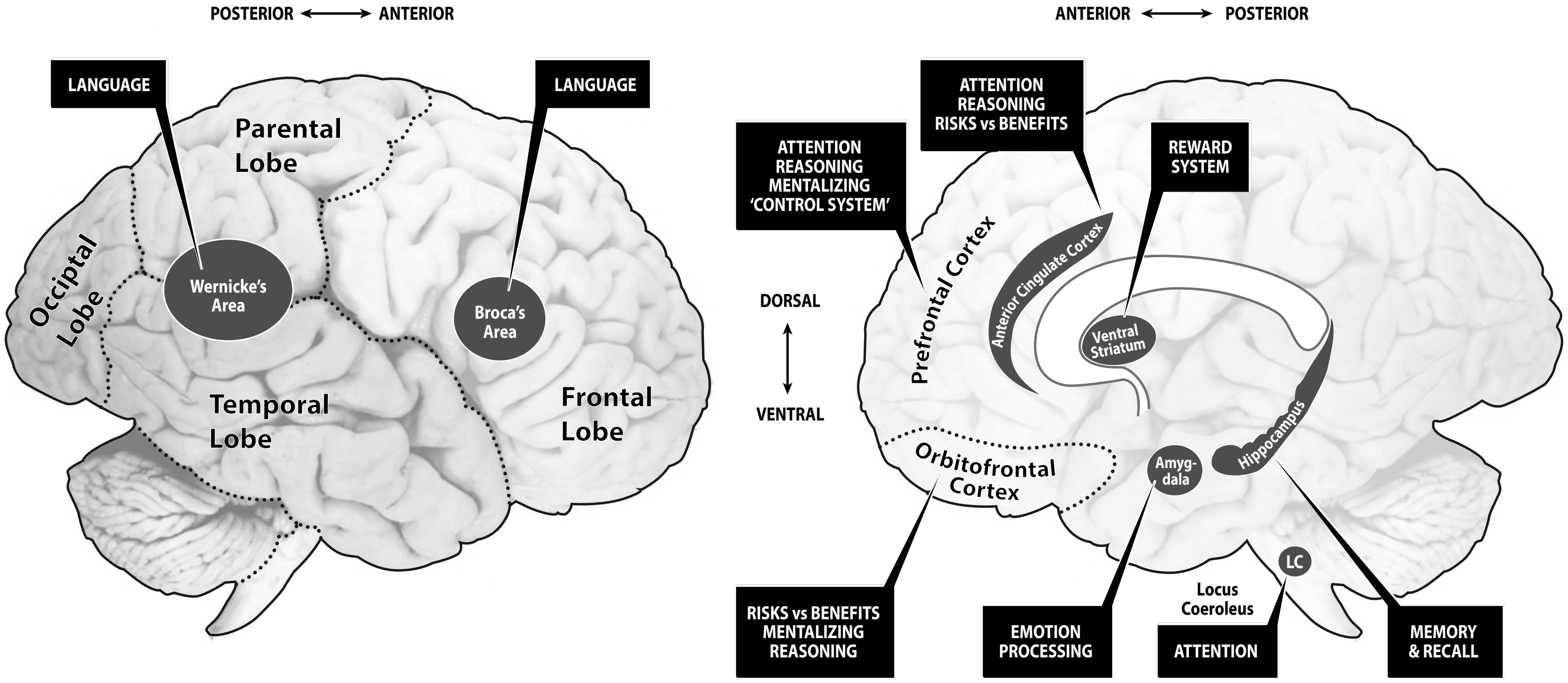

POSTERIOR ⟷ ANTERIOR
LANGUAGE
LANGUAGE
Parental Lobe
Occiptal Lobe
Wernicke's Area
Broca's Area
Temporal Lobe
Frontal Lobe
ANTERIOR ⟷ POSTERIOR
ATTENTION REASONING RISKS vs BENEFITS
ATTENTION REASONING MENTALIZING 'CONTROL SYSTEM'
REWARD SYSTEM
Prefrontal Cortex
Anterior Cingulate Cortex
Ventral Striatum
DORSAL
VENTRAL
Orbitofrontal Cortex
Amyg-dala
Hippocampus
LC
Locus Coeroleus
RISKS vs BENEFITS MENTALIZING REASONING
EMOTION PROCESSING
ATTENTION
MEMORY & RECALL
